# Research ethics with real-world data (RWD) on COVID-19 infections: the unCoVer study: Silvia Riva

**DOI:** 10.1093/eurpub/ckac130.060

**Published:** 2022-10-25

**Authors:** S Riva, Z Kabir, N Biscoe, G O'Sullivan, G Soriano, J Penalvo

**Affiliations:** 1 St. Mary's University, Twickenham, London, UK; 2 University College of Cork, Cork, Ireland; 3 Institute of Tropical Medicine, Antwerp, Belgium

## Abstract

**Issue:**

The aim of the Horizon 2020 unCoVer project (Unravelling Data for Rapid Evidence-Based Response to COVID-19) is to coordinate research expertise in utilising Real World Data (RWD) to investigate the underlying risk factors for COVID-19 infection and severity, the effectiveness of treatments and the impact on health systems. RWD is particularly useful in a dynamic health context as it is relevant, timely, and more ecologically valid. Pooling clinical databases and integrating epidemiological principles and powerful biostatistical tools optimises resources and fully exploits routinely-collected data.

**Description of the problem:**

RWD sharing poses new practical and ethical challenges to research. The unCoVer network has developed a federated data platform to access diverse databases for advanced analytics. This data access process entails GDPR, and regulatory and ethical nuances. The use of large-scale data from heterogeneous sources across multiple jurisdictions for research purposes presents a complex systems challenge.

**Effects & Lessons:**

A dedicated team of unCoVer network members is responsible for addressing these challenges. Here, we describe the ethical and regulatory aspects of RWD sources, the role of the Data Protection Authorities and the Data Protection External Authority Board (DP-EAB) of the Uncover project, and the documentation involved, including a data processing agreement and a data transfer agreement. We provide an overview of the main principles for sharing RWD whilst maintaining integrity and security and how this translates into procedures to protect the rights, security, and well-being of human research participants. This represents a practical framework for researchers.

**Key messages:**

- Sharing RWDs presents new practical and ethical challenges for research where large-scale datasets from heterogeneous sources across multiple jurisdictions must be arranged in a structured manner.
- Using the Uncover project framework, heterogeneous data can be shared and harmonized in a standard manner. This framework can be used in future RWD projects to generate real-world evidence.

